# Viscoelastic Response of Double Hydrophilic Block Copolymers for Drug Delivery Applications

**DOI:** 10.3390/polym17131857

**Published:** 2025-07-02

**Authors:** Achilleas Pipertzis, Angeliki Chroni, Stergios Pispas, Jan Swenson

**Affiliations:** 1 Department of Physics, Chalmers University of Technology, 41296 Gothenburg, Sweden; jan.swenson@chalmers.se; 2 Theoretical and Physical Chemistry Institute, National Hellenic Research Foundation, 48 Vassileos Constantinou Ave., 11635 Athens, Greece; angelikechrone@gmail.com (A.C.); pispas@eie.gr (S.P.)

**Keywords:** double hydrophilic block copolymer, densely grafted architecture, rheology, viscoelastic response

## Abstract

This study investigates the mechanical properties of double hydrophilic block copolymers (DHBCs) based on poly[oligo(ethylene glycol) methacrylate] (POEGMA) and poly(vinyl benzyl trimethylammonium chloride) (PVBTMAC) blocks by employing small amplitude oscillatory shear (SAOS) rheological measurements. We report that the mechanical properties of DHBCs are governed by the interfacial glass transition temperature (*T*_g_^inter^), verifying the disordered state of these copolymers. An increase in zero shear viscosity can be observed by increasing the VBTMAC content, yielding a transition from liquid-like to gel-like and finally to an elastic-like response for the PVBTMAC homopolymer. By changing the block arrangement along the backbone from statistical to sequential, a distinct change in the viscoelastic response is obvious, indicating the presence/absence of bulk-like regions. The tunable viscosity values and shear-thinning behavior achieved through alteration of the copolymer composition and block arrangement along the backbone render the studied DHBCs promising candidates for drug delivery applications. In the second part, the rheological data are analyzed within the framework of the classical free volume theories of glass formation. Specifically, the copolymers exhibit reduced fractional free volume and similar fragility values compared to the PVBTMAC homopolymer. On the contrary, the activation energy increases by increasing the VBTMAC content, reflecting the required higher energy for the relaxation of the glassy VBTMAC segments. Overall, this study provides information about the viscoelastic properties of DHBCs with densely grafted macromolecular architecture and shows how the mechanical and dynamical properties can be tailored for different drug delivery applications by simply altering the ratio between the two homopolymers.

## 1. Introduction

Copolymers are made from two or more different monomers [1,2]. The advantage of copolymers compared to homopolymers is that it is much easier to tailor their properties. For instance, their balance between hydrophilicity and hydrophobicity, degradation rate, mechanical strength and solubility can easily be optimized for their specific target applications. Particularly promising polymers to use for the fabrication of copolymers are so-called bottlebrush polymers, which consist of a linear backbone with densely grafted side chains extending outward, creating a highly extended and bulky structure [3,4,5,6,7,8,9,10]. The bottlebrush polymers have attracted considerable attention owing to their highly tunable physical and chemical properties, which depend on side chain length, backbone length, and grafting density [3,4,5,6,7,8,9,10]. In comb polymers, side chains act as a solvent, diluting backbone concentration—a phenomenon known as dynamic tube dilution [6,11,12,13]. This effect results in a low viscosity and a reduced rubbery plateau. These properties make bottlebrush block copolymers promising for applications in drug delivery, energy storage, nanostructures, catalysis, and 3D printing [14,15,16,17,18,19,20,21,22,23,24,25]. In the case of copolymers for drug delivery applications, it is, for instance, possible to tune their properties to optimize drug release, targeting molecules and biocompatibility [26,27].

Several studies [1,2,28] have indicated that the block arrangement along the polymer backbone significantly influences viscoelastic and mechanical properties, with sequential copolymers generally exhibiting enhanced phase separation and mechanical strength compared to their statistical counterparts. In contrast, statistical or random copolymers lack long-range compositional order, typically exhibiting homogeneous amorphous morphologies. The absence of microphase separation in these materials results in more viscous and less elastic behavior, with lower shear moduli. Therefore, the block arrangement is a critical parameter in tuning the mechanical and structural performance of copolymeric materials [1,2,28].

The viscoelastic response of polymeric formulas plays a critical role in drug delivery applications [29,30,31,32,33,34]. The ideal viscoelastic properties ensure a balance between elasticity (for mechanical stability) and viscosity (for flow and processability). One key requirement is a shear-thinning behavior (i.e., reduced viscosity at higher frequencies), which enables ease of injection or spreading [29,30,31]. The viscoelastic properties also affect the diffusion mechanism, which is critical for obtaining a stimuli-responsive release profile. Another vital requirement of the polymeric material is a high degree of biodegradability and biocompatibility, without altering the release characteristics [33,34]. Therefore, tailoring the viscoelastic behavior through the use of copolymers with varied compositions is essential to meet the specific needs for drug delivery applications.

Double hydrophilic block copolymers (DHBCs), composed of two chemically distinct water-soluble blocks, are important in polymer science, pharmacy, biophysics and biochemistry [35,36,37,38]. They offer an alternative option to traditional amphiphilic block copolymers and self-assemble in aqueous conditions in response to changes in ionic strength, temperature, pH, or complexation with specific (bio)molecules. DHBCs with charged blocks, known as polyelectrolytes, are promising candidates for biomacromolecule delivery via electrostatic complexation [39]. For enhancing water solubility and stability in aqueous media, a neutral block, such as poly(ethylene glycol) or poly(oligo(ethylene glycol) methacrylate) (POEGMA), is used. A recent review article by Singh et al. provides an overview of the synthesis and biomedical applications of POEGMA-based materials [33]. Diblock and statistical copolymers consisting of POEGMA and poly(vinyl benzyl trimethylammonium chloride) (PVBTMAC) have been successfully synthesized using a reversible addition−fragmentation chain transfer (RAFT) polymerization process [37,38]. Recent research studies have focused on these copolymers’ abilities to form electrostatic complexes with hydrophilic magnetic nanoparticles, short DNA and negatively charged proteins (i.e., insulin) [37,38]. Additionally, the relaxation dynamics and the self-assembly of these DHBCs were investigated under dry conditions [40]. It was shown that the weak segregation strength between the two hydrophilic blocks results in homogeneous dynamics that are governed by the interfacial glass transition temperature (*T*_g_^inter^) [40].

Herein, the previously described block and random DHBCs were studied by means of rheological measurements. We report that the PVBTMAC homopolymer exhibits an elastic response up to high temperatures, reflecting its strong (i.e., rigid) nature. The effect of the VBTMAC block on the zero shear viscosity is presented, and the rheological data are discussed with respect to classical free volume theories of glass formation. The values of fractional free volume, thermal expansion coefficient, fragility, and apparent activation energy at the glass transition temperature (*T*_g_) are reported and compared with those of the PVBTMAC parent homopolymer. The results indicate that a synergistic combination of hydrophilicity, biodegradability and tunable rheological properties (such as shear-thinning behavior and viscosity) can be achieved through optimization of the copolymer composition and block arrangement along the backbone, rendering the studied DHBCs promising candidates for drug delivery applications.

## 2. Materials and Methods

### 2.1. Synthesis

**Figure 1:**
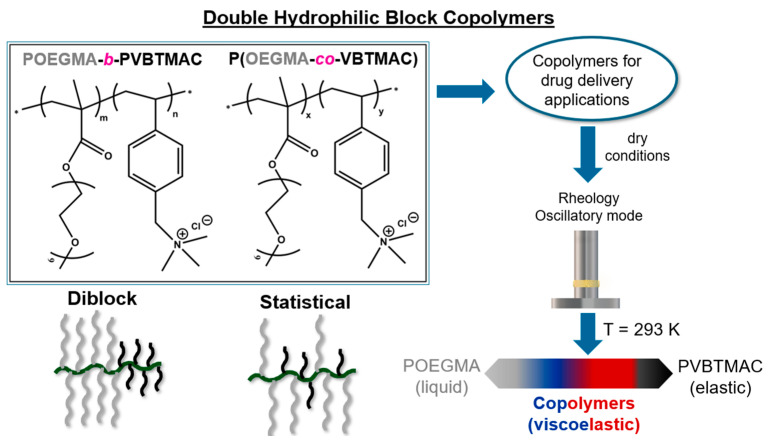
Chemical structure of the dried block and random DHBCs along with a schematic of the block sequence along the backbone and a description of the motivation of this study.

The synthetic procedure and molecular characterization of the studied DHBCs are highlighted in previous studies [37,38]. Specifically, the charged DHBCs with densely grafted macromolecular architecture were prepared by RAFT polymerization, an advantageous method for controlling the molar mass (*M*_w_) and achieving polydispersity values close to unity [41,42]. The chemical structure of the investigated DHBCs is depicted in Figure 1.

### 2.2. Rheology

Rheological measurements into the linear viscoelastic regime (LVR) can provide quantitative insight into the viscoelastic response of the studied polymeric materials [43]. An MCR-302 twin mode rheometer by Anton Paar was used for the identification of the viscoelastic properties of grafted copolymers under dry conditions. Measurements were made with the environmental test chamber as a function of temperature by using the heating system (P-PTD200). The samples were prepared on the lower rheometer plate with a diameter of 8 mm. Specifically, the upper plate was brought into contact, and the sample thickness was adjusted accordingly. Typically, the gap between plates ranged between 0.3 mm and 0.6 mm among the investigated DHBCs. At each temperature the linear viscoelastic region of the copolymers was determined by performing dynamic strain sweeps from 0.01 to 50% (the upper limit varies regarding the state of the material) at *ω* = 10 rad·s^−1^, as shown in Figure S1. Subsequently, isothermal frequency sweeps in an angular frequency range of 0.1 < ω < 100 rad·s^−1^ were carried out in temperature steps of 10 K by employing the extracted strain amplitude of the dynamic strain sweep. Before each isothermal measurement, a thermal stabilization of 20 min was employed to ensure thermal equilibrium. Data collected under the minimum torque of the instrument were excluded. Master curves were constructed by using the principle of time–temperature superposition (*tT*s). For estimating the zero shear viscosity, we used an extrapolation procedure for the data that do not reach the Newtonian limit at low frequencies. Empirical models can be utilized to fit the complex viscosity response and extrapolate to low frequencies to estimate the value of zero shear viscosity, *η*_0_. Specifically, the Carreau–Yasuda (CY) model is given by the following equation [44,45]:

(1) $$\eta^{*}\left(\omega \right)=\eta_{0}{\left[1+{\left(\lambda \omega \right)}^{\alpha }\right]}^{\frac{n-1}{\alpha }},$$

where *λ* is the relaxation time, α indicates the width of the transition region between Newtonian and power-law behavior, and *n* is the power-law index (i.e., a measure of the shear-thinning nature of a polymer melt). All these parameters can be obtained by fitting the measured data. Another empirical model that can be used for fitting the viscosity data is the modified Cross (MC) model, according to [46]

(2) $$\eta^{*}\left(\omega \right)=\frac{\eta_{0}}{1+{\left[C_{0}\omega \right]}^{1-n}},$$

where *C*_0_ and *n* can be extracted by fitting the data to Equation (2).

## 3. Results and Discussion

Quantitative insight into the viscoelastic response of the dried DHBCs can be obtained through small amplitude oscillatory shear (SAOS) rheological measurements. The effect of temperature under isochronal conditions (i.e., at a fixed angular frequency of 10 rad·s^−1^) on the mechanical properties of the dried copolymers is depicted in Figure S2. The PVBTMAC homopolymer exhibits elastic behavior up to extremely high temperatures, indicating its rigid/strong nature. The temperature dependence of the loss factor verifies the existence of two *T*_g_s, associated with the backbone and side chain vitrification [40]. The glass transition temperatures of the PVBTMAC homopolymer and the statistical copolymers obtained from rheological measurements are higher compared to those determined by calorimetric and dielectric techniques [40]. This discrepancy arises primarily from two factors: (i) differences in the characteristic timescales/frequencies of the methods (i.e., the dielectric *T*_g_ is typically defined at a relaxation time of *τ* = 100 s) and (ii) the rheological *T*_g_, herein determined at the peak of the loss tangent (tan*δ*), reflects the midpoint of the transition from the glassy modulus (*G*_g_) to the rubbery or entanglement plateau (*G*_e_). Consequently, the rheological *T*_g_ corresponds to the end region of the glass transition. Regarding the statistical copolymers, at a fixed temperature, they exhibit reduced shear moduli that are attributable to the influence of the soft OEGMA segments. Additionally, the viscoelastic response is governed by *T*_g_^inter^, verifying their disordered and well-mixed state, as observed by X-ray diffraction [40].

**Figure 2:**
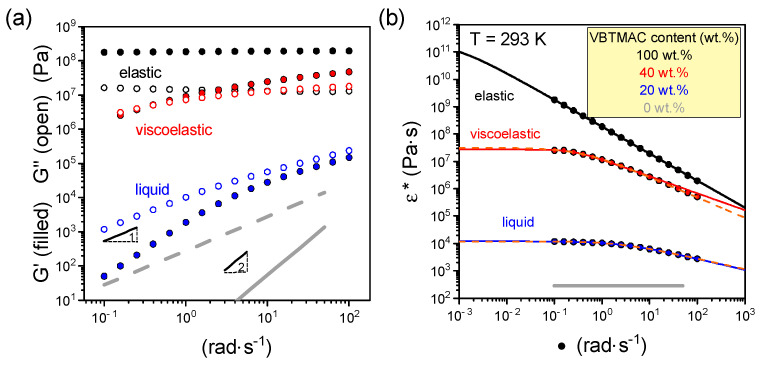
Angular frequency dependence of (**a**) the storage (filled symbols) and loss (open symbols) shear moduli and (**b**) complex viscosity for P(OEGMA_80_-*co*-VBTMAC_20_) (blue symbols) and P(OEGMA_60_-*co*-VBTMAC_40_) (red symbols) DHBCs and their respective homopolymers POEGMA (gray symbols) and PVBTMAC (black symbols) at a temperature of 293 K. Lines with slopes 1 and 2 are also shown in (**a**). The solid and dashed lines in (**b**) represent fits by Equation (1) and Equation (2), respectively.

The dynamic frequency sweeps into the linear viscoelastic response under isothermal conditions (i.e., 293 K) for the statistical copolymers with various compositions are shown in Figure 2 and Figure S3.

Initially, the dynamic frequency sweep reveals that the PVBTMAC homopolymer exhibits a strong nature and elastic response (*G*′ >> *G*″ ~ *ω*^0^). In going to the statistical copolymer containing 40 wt.% VBTMAC, a viscoelastic response, characterized by comparable and frequency-dependent storage (*G*′) and loss (*G*″) moduli, can be observed. By further decreasing the VBTMAC content to 20 wt.%, a liquid-like response (*G*′ ~ ω^2^ and *G*″ ~ ω^1^) is evident. In the latter copolymer, the complex viscosity ($\eta^{*}=\sqrt{{G^{\prime }}^{2}+{G^{\prime \prime }}^{2}}/\omega$), exhibits a plateau at lower angular frequencies, from which the zero shear viscosity, *η*_0_, can be extracted. For extracting the zero shear viscosity, the measured data were fitted/modeled with two different empirical models, as detailed in the experimental section. The fitting parameters are provided in Table 1. In addition, the dynamic frequency data of the statistical copolymers are compared and contrasted with those found at PVBTMAC homopolymer under iso-*T*_g_ temperatures (i.e., *T*_g_ + 30 K) in Figure S4. The iso-*T*_g_ comparison reflects the changes in viscoelastic response from elastic to viscoelastic and liquid response by decreasing the VBTMAC content, quantitatively verifying the observations at ambient temperature.

Concerning the statistical copolymers, the relaxation time, λ, increases by increasing the concentration of VBTMAC glassy blocks. The extracted values of the power law index, *n*, imply a shear-thinning behavior of the copolymer melts that becomes stronger by increasing VBTMAC content. For the copolymer with 40 wt.% VBTMAC and the PVBTMAC homopolymer, the values of zero shear viscosity extracted from the MC model are slightly higher than the ones obtained from the CY model.

**Figure 3:**
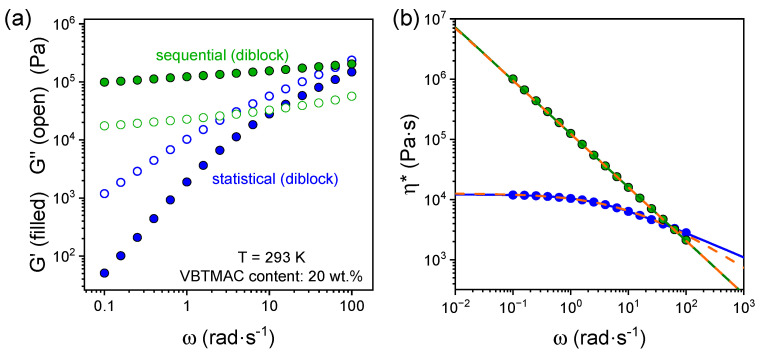
Angular frequency dependence of (**a**) the storage (filled symbols) and loss (open symbols) shear moduli and (**b**) complex viscosity for the sequential (green) and statistical (blue) DHBCs with 20 wt.% of VBTMAC. The solid and dashed lines in (**b**) represent fits by Equations (1) and (2), respectively.

The effect of block arrangement along the backbone can be discussed with respect to Figure 3 and Table 1, comparing the dynamic frequency sweep data of the sequential and statistical copolymer with a VBTMAC content of 20 wt.%.

The sequential arrangement of VBTMAC glassy blocks yields an elastic response (*G*′ >> *G*″ and *G*′, *G*′ ~ *ω*^0^), compared to the liquid-like response of the statistical copolymer, indicating the presence of larger and continuous bulk-like VBTMAC regions, despite the fact that both specimens are in the disordered state, as evidenced in our previous study [40]. Parenthetically, a reduced change in heat capacity for *T*_g_^inter^ was evidenced for the sequential copolymer compared to the statistical, verifying the aforementioned observations [40]. The determination of the zero shear viscosity is challenging and inaccurate for the sequential copolymer, due to its predominantly elastic response, even at elevated temperatures (see Figures S5 and S6), hindering the low-frequency plateau in the viscosity data. To mitigate this challenge, the viscosity data were fitted with the empirical models of Equations (1) and (2), yielding a higher value of zero shear viscosity by approximately three orders of magnitude compared to the corresponding statistical copolymer. This indicates that the block arrangement along the copolymer backbone plays a crucial role in tailoring the viscoelastic response of the studied DHBCs.

**Figure 4:**
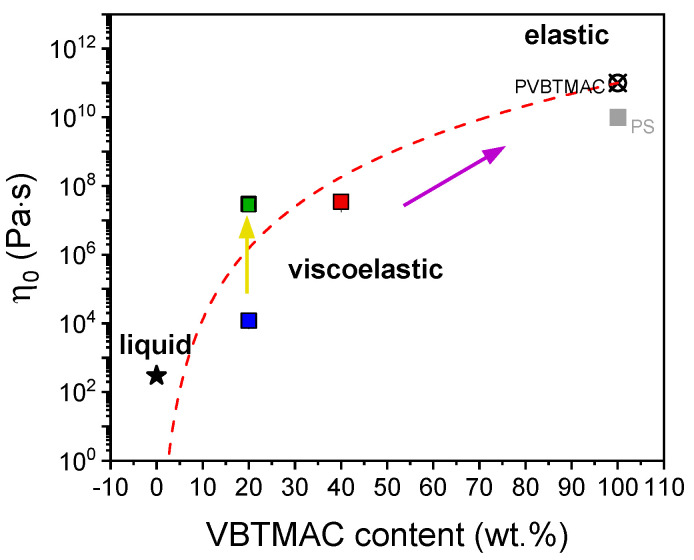
Zero shear viscosity, *η*_0_, as a function of the VBTMAC weight fraction in DHBCs and the corresponding parent homopolymers at ambient temperature. The zero shear viscosity for polystyrene (PS) is also included for comparison and taken from Ref. [47]. The yellow and purple arrows indicate the effect of block arrangement across the backbone and the impact of VBTMAC content on the zero shear viscosity, respectively. The red dashed line is a guide for the eye.

The extracted values for zero shear viscosity are summarized in Figure 4 at ambient temperature.

The zero shear viscosity values can be dictated by changing (i) the copolymer’s composition and (ii) the block arrangement along the backbone. This is advantageous for the use of these copolymers in drug delivery applications. The dynamic frequency sweeps for the statistical copolymers are presented at different temperatures in Figure S7, along with the temperature dependence of the extracted power law index. The latter increases by increasing temperature, implying less shear thinning behavior, as evident from the flattening of the viscosity curves. The shear-thinning behavior of the studied polymeric formulations can be tailored by varying (i) temperature, (ii) composition, and (iii) block arrangement along the polymer backbone, highlighting their potential for biomedical applications.

**Figure 5:**
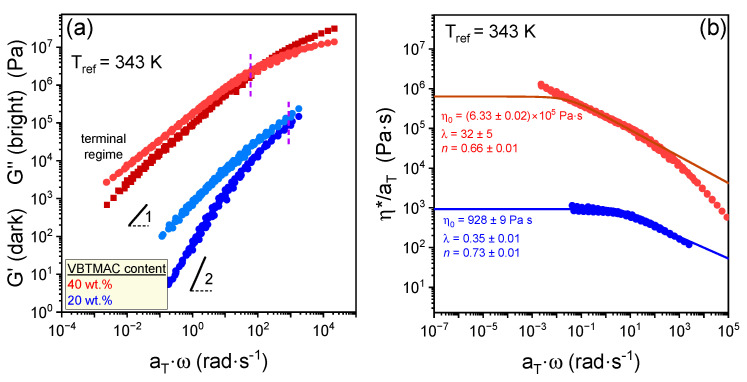
Superimposed master curves of (**a**) the storage (dark symbols) and loss (bright symbols) shear moduli and (**b**) complex viscosity constructed by employing the principle of *tT*s for the statistical DHBCs with 20 wt.% (blue symbols) and 40 wt.% (red symbols) VBTMAC content, as indicated. Lines with slopes 1 and 2 are also shown in (**a**). The solid lines represent fits by Equation (2) in (**b**).

Detailed information about the viscoelastic response of the studied copolymers can be obtained through the construction of master curves by employing the *tT*s principle, which involves the horizontal shifting of dynamic frequency sweeps, as shown in Figure 5. The original data of the storage and loss moduli are presented in Figures S8 and S9.

**Figure 6:**
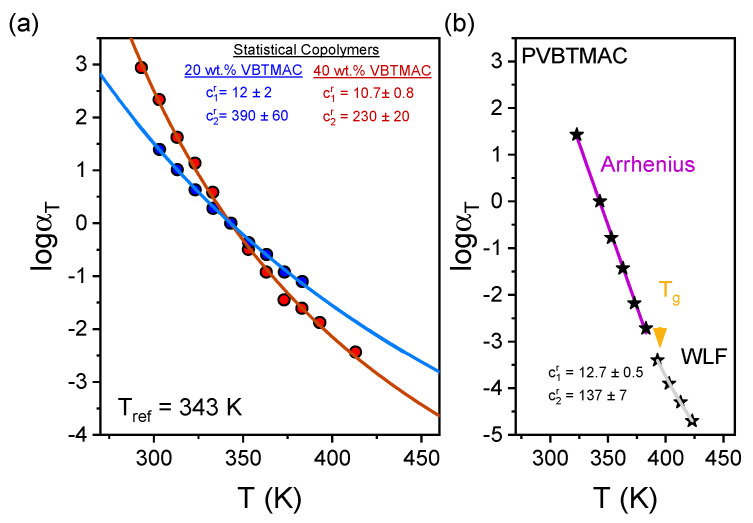
Temperature dependence of the horizontal shift factors for (**a**) statistical DHBCs and (**b**) the PVBTMAC homopolymer.

For the copolymer containing 20 wt.% VBTMAC, a well-defined terminal regime is observed, confirming its liquid- or melt-like behavior. The *G*′(*ω*) does not follow the ideal terminal relaxation (G′ ~ ω^2^), reflecting mainly the ionic interactions arising from the charged VBTMAC side groups. These ionic interactions persist into the melt state, influencing its terminal relaxation. In contrast, the copolymer with 40 wt.% VBTMAC maintains a viscoelastic response (G′ ~ ω) even at high temperatures (i.e., low frequencies), highlighting the influence of the glassy VBTMAC segments and the presence of strong ionic interactions. As depicted in Figure 5 and Figure S2, the absence of large rubbery plateaus is attributable to the low molar masses and the short side chain lengths that distinctly reduce the entanglements. Figure 5b presents the normalized complex viscosity, adjusted using horizontal shift factors, as a function of normalized angular frequency. This representation of the master curves for the statistical copolymers confirms the viscosity variation with copolymer composition. Simultaneously, the extracted values of the power law index, by employing the MC model, indicate that both statistical copolymers display comparable levels of shear-thinning behavior. The temperature dependencies of the extracted horizontal shift factors for the statistical DHBCs and the PVBTMAC homopolymer are depicted in Figure 6. The original data of the frequency sweeps for the PVBTMAC homopolymer are presented in Figure S10.

The results can now be discussed in terms of classical free volume theories of glass formation [43]. As shown in Figure 6, the extracted horizontal shift factors, *α*_T_, were fitted according to the Williamas–Landel–Ferry (WLF) equation [48]:

(3) $$\log\alpha_{T}=-\frac{{c_{1}}^{r}\left(T-T^{r}\right)}{{c_{2}}^{r}\left(T-T^{r}\right)},$$

where *c*_1_^r^ and *c*_2_^r^ are empirical parameters at the reference temperature, that is, *T*_r_ = 343 K, for the studied statistical DHBCs. For the PVBTMAC homopolymer, a transition from WLF to Arrhenius-type temperature dependence is observed in the vicinity of *T*_g_ (~383 K), corresponding to the vitrification of the polystyrene backbone. According to the theory, the empirical parameters can be calculated at *T*_g_: c_1_^g^ = *c*_1_^r^ *c*_2_^r^/(*c*_2_^r^ + *T*_g_ − *T*_r_) and *c*_2_^g^ = *c*_2_^r^ +*T*_g_ − *T*_r_, and then the fractional free volume, f(*T*_g_), and the thermal expansion coefficient of free volume, α_f_, can be extracted, according to

(4) $$f(T_{g})=\frac{1}{2.303c_{1}^{g}},$$

(5) $$\alpha_{f}=\frac{f\left(T_{g}\right)}{c_{2}^{g}}.$$

Furthermore, the fragility or steepness index, m*, and apparent activation energy, *E*_g_, can be estimated from [49,50]

(6) $$m^{*}=\frac{c_{1}^{g}T_{g}}{c_{2}^{g}},$$

(7) $$E_{g}=2.303R\frac{c_{1}^{g}}{c_{2}^{g}}T_{g}^{2}.$$

**Figure 7:**
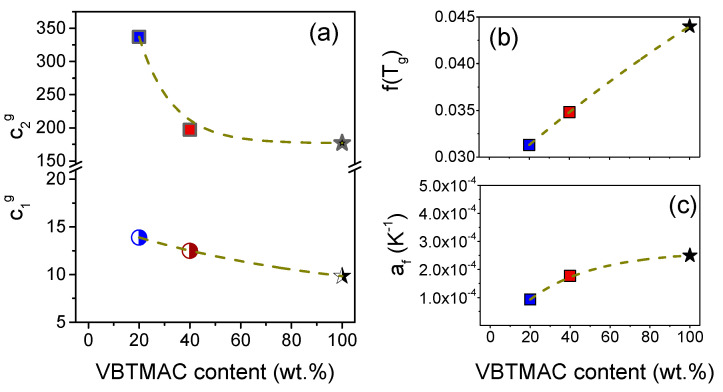
(**a**) WLF coefficients, *c*_1_^g^ (semi-filled symbols) and *c*_2_^g^ (filled symbols), (**b**) fractional free volume and (**c**) thermal expansion coefficient of free volume at *T*_g_, determined through rheology, plotted as a function of the VBTMAC content. Dashed lines are guides for the eye.

**Figure 8:**
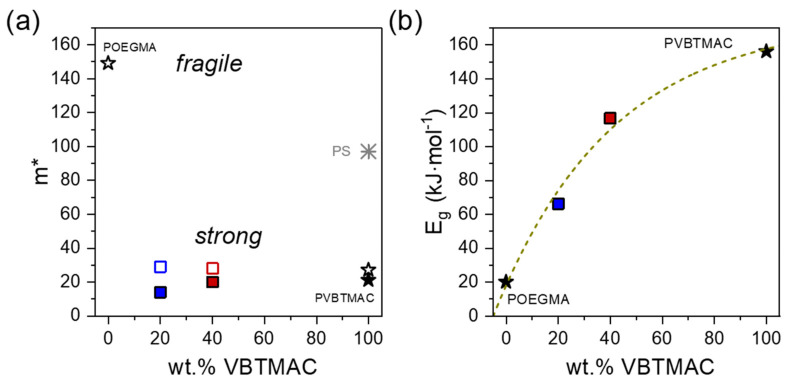
(**a**) Fragility and (**b**) apparent activation energy as a function of the VBTMAC weight fraction for the copolymers (squares) and their parent blocks (stars). Fragility data from dielectric measurements, taken from Ref. [40], are also included along with fragility data for PS taken from Ref. [51].

The estimated parameters are summarized in Table 2 and can be discussed with respect to Figure 7 and Figure 8.

As depicted in Figure 7, the *c*_1_^g^ and *c*_2_^g^ parameters are dependent on the copolymer composition, leading to differences in the fractional free volume and thermal expansion coefficient according to Equations (4) and (5). Specifically, the fractional free volume increases by increasing the VBTMAC content. The reason for this is most likely that the bulky ionic side chains of the VBTMAC blocks increase the segmental rigidity and steric hindrance, leading to packing frustration. This also results in a slight increment of the thermal expansion coefficient. Importantly, the *c*_2_^g^ of PVBTMAC is approximately three times larger compared to polystyrene (PS) [51], thereby reducing fragility values, as discussed below in relation to Figure 8.

The PVBTMAC homopolymer exhibits one of the lowest fragility values ever reported for common polymers (see Figure S11), reflecting a *superstrong* behavior [52,53]. The rheology-derived values are in line with those found from dielectric measurements [40]. This low value of fragility indicates a less cooperative character of the structural relaxation, meaning that fewer neighboring segments cooperate in the structural relaxation. Specifically, the PVBTMAC homopolymer exhibits distinctly reduced fragility values compared to the linear polystyrene [43,51]. A similar trend has been observed in the family of poly(*p*-alkyl methacrylates), where a change from a “fragile” (*m* = 92) to a “strong” (*m* = 36) liquid occurs only by increasing the length of the side chain with the addition of a methylene unit (see Figure S12) [54,55]. Specifically, the PVBTMAC homopolymer exhibits a slightly lower value of fragility than poly(*p*-alkyl methacrylates) with side chain lengths, *p*, bearing 1 < *p* < 18 methylene units [55,56,57] and the poly(*p*-phenylene) homopolymer [58] bearing side chain length with 8 methylene units, despite the higher *T*_g_ of PVBTMAC. Therefore, the superstrong nature of the PVBTMAC homopolymer is probably driven mainly by its side chain length and its high *T*_g_ that is mainly dictated by the strong electrostatic interactions taking place along its side chains.

Concerning the statistical copolymers, a similar *superstrong* behavior can be observed. Anticipatedly, as shown in Figure 8b, the apparent activation energy strongly increases by increasing the VBTMAC content, reflecting the stiff nature of VBTMAC segments and the higher amount of energy that is required for their segmental relaxation. This *superstrong* nature of the studied polymeric formulations may be a valuable attribute for their use in biomedical applications.

To sum up, this study offers a comprehensive quantitative analysis of the viscoelastic behavior of both sequential and statistical DHBCs. The findings demonstrate that the viscoelastic response of these systems can be precisely modulated by altering the copolymer composition and the arrangement of the hydrophilic blocks, as well as by adjusting the environmental temperature. Variations in block architecture (e.g., sequential versus statistical copolymers) lead to distinct molecular interactions and microphase behavior, which in turn govern the macroscopic rheological properties such as storage moduli (*G*′) and loss moduli (*G*″), and thus the viscosity.

These tunable viscoelastic properties are critical for drug delivery applications, where formulation performance often depends on the mechanical adaptability, injectability and controlled release capability of the carrier matrix. For instance, a copolymer with a high shear moduli may provide better mechanical integrity at the site of delivery, while a reduced viscosity or gel-like response at physiological temperatures can enable sustained drug release. The ability to systematically tailor these rheological characteristics makes DHBCs versatile components for designing smart and responsive drug delivery systems.

## 4. Conclusions

Herein, we have investigated the mechanical properties of block and random DHBCs using rheological measurements. We report that the mechanical properties are governed mainly by the interfacial *T*_g_. Depending on the VBTMAC content and block arrangement across the backbone, the macroscopic mechanical properties change from elastic-like to liquid-like. Specifically, the PVBTMAC homopolymer exhibits an elastic response up to high temperatures, reflecting its strong nature. A viscoelastic response is evident for the statistical copolymer with 40 wt.% VBTMAC. By further decreasing the VBTMAC to 20 wt.%, the statistical copolymer exhibits a liquid-like response. Overall, the zero shear viscosity, determined by fitting the measured data with two empirical models (i.e., the Carreau–Yasuda and modified Cross models), exponentially increases by increasing the VBTMAC content. This behavior reflects the influence of the glassy VBTMAC segments and the presence of strong ionic interactions arising from the charged VBTMAC side groups. Furthermore, for the 20 wt.% VBTMAC content, the change in block arrangement of the backbone from statistical to sequential results in a transition from liquid-like to elastic-like behavior, accompanied by an increase in zero shear viscosity.

In terms of classical free volume theories [43], we report that the copolymers exhibit reduced fragility reminiscent of that found in the PVBTMAC parent block. Importantly, the PVBTMAC homopolymer exhibits 5-fold lower fragility than the one found for polystyrene, reflecting its *superstrong* nature. The above observations are in line with previously reported dielectric results [40]. A *superstrong* behavior is also evident for the statistical copolymers, reflecting the impact of the glassy VBTMAC segments and their miscible state. To conclude, this study illustrates the tunability of the viscosity and shear-thinning behavior through systematic variation in (i) the brush copolymer composition, (ii) the block sequence along the polymer backbone, and (iii) the temperature, which furthermore demonstrates the potential of the investigated copolymers for advanced biomedical applications such as drug delivery.

## Figures and Tables

**Table 1:** Zero shear viscosity and fitting parameters of CY and MC models.

VBTMAC (wt.%)	*η*_0_ (Pa·s)	*λ* (s)	*n*	*α*
Carreau–Yasuda (CY) model				
20 (sequential)	(3 ± 1) × 10^7^	580 ± 10	0.11 ± 0.02	2 (fixed)
20 (statistical)	11,970 ± 30	0.43 ± 0.01	0.59 ± 0.01	0.91 ± 0.03
40 (statistical)	(2.68 ± 0.08) × 10^7^	3.8 ± 0.3	0.38 ± 0.02	2.0 ± 0.4
100	(2 ± 2) × 10^11^	2000 ± 1000	0.015 ± 0.002	0.99 ± 0.2
Modified Cross (MC) model				
20 (sequential)	(3 ± 1) × 10^7^	1800 ± 900	0.11 ± 0.02	-
20 (statistical)	11,970 ± 30	0.43 ± 0.01	0.61 ± 0.01	-
40 (statistical)	(3.13 ± 0.08) × 10^7^	2.65 ± 0.4	0.25 ± 0.04	-
100	(2.47 ± 0.09) × 10^11^	1490 ± 60	0.015 ± 0.002	-

**Table 2:** Rheological *T*_g_ and WLF parameters, fractional free volume, thermal expansion coefficient, fragility along with apparent activation energy at *T*_g_ for DHBCs and PVBTMAC homopolymer.

Sample Code	*T*_g_ (K) *	*c*_1_^g^	*c*_2_^g^ (K)	f(*T*_g_)	α_f_ (K^−1^)	m*	*E*_g_ (kJ·mol^−1^)
P(OEGMA_80_-*co*-VBTMAC_20_)	290	13.89	337	0.0313	9.29 × 10^−5^	12	66.4
P(OEGMA_60_-*co*-VBTMAC_40_)	310	12.49	197	0.0348	1.77 × 10^−4^	20	116.7
PVBTMAC	383	9.83	177	0.044	2.5 × 10^−4^	21	156.0

* *T*_g_ determined from rheology is higher compared to the dielectric one due to the fact that the dynamic frequency sweeps are performed at *ω* = 10 rad·s^−1^.

## Data Availability

Data will be made available on request.

## Supplementary Materials

The following supporting information can be downloaded at https://www.mdpi.com/article/10.3390/polym17131857/s1, Figure S1: Strain sweep test; Figure S2: Temperature ramp of shear storage and loss modulus, along with the loss factor curves; Figure S3: Angular frequency sweeps for the studied statistical copolymers and their parent homopolymers; Figure S4: Angular frequency sweeps at iso-*T*_g_ temperatures; Figure S5: temperature ramps for diblock and statistical copolymer with 20 wt.% of VBTMAC; Figure S6: Angular frequency sweeps for diblock copolymer at two temperatures; Figure S7: Complex viscosity curves and the extracted power law index at different temperatures; Figures S8 and S9: Original dynamic frequency sweep data for the statistical copolymers; Figure S10: Original data for the PVBTMAC homopolymer; Figures S11 and S12: Comparison of fragility values with the literature-reported data.

## Abbreviations

The following abbreviations are used in this manuscript:

DHBCs	double hydrophilic block copolymers
POEGMA	poly[oligo(ethylene glycol) methacrylate]
PVBTMAC	poly(vinyl benzyl trimethylammonium chloride)
SAOS	small amplitude oscillatory shear
*T*_g_^inter^	interfacial glass transition temperature
RAFT	reversible addition–fragmentation chain transfer
*T*_g_	glass transition temperature
LVR	linear viscoelastic regime
CY	Carreau–Yasuda model
MC	modified Cross model
|*G**|	complex shear moduli
*G*′	shear storage moduli
*G*″	shear loss moduli
η_0_	zero shear viscosity
tTs	time–temperature superposition
WLF	Williamas–Landel–Ferry
α_T_	horizontal shift factor
f(*T*_g_)	fractional free volume at *T*_g_
α_f_	thermal expansion coefficient of free volume at *T*_g_
*m**	fragility
*E*_g_	apparent activation energy
PS	polystyrene
