# Supplementary material for: Viscoelastic Response of Double Hydrophilic Block Copolymers for Drug Delivery Applications
Source: Polymers (Basel). 2025 Jul 2;17(13):1857. doi: 10.3390/polym17131857 (PMC12252078; doi:10.3390/polym17131857)
Supplement: Supplementary file 1 [file polymers-17-01857-s001.zip › polymers-3707511-supplementary.pdf]

Supporting Information

Viscoelastic Response of Double Hydrophilic Block Copolymers for Drug Delivery Applications

Achilleas Pipertzis,[1*] *Angeliki Chroni,*[2] *Stergios Pispas,*[2] *Jan Swenson*[1]

[1]*Department of Physics, Chalmers University of Technology, 41296, Gothenburg, Sweden*

[2]*Theoretical and Physical Chemistry Institute, National Hellenic Research Foundation, 48 Vassileos Constantinou Ave., 11635 Athens.*

Correspondence: Achilleas Pipertzis

Email: achilleas.pipertzis@chalmers.se

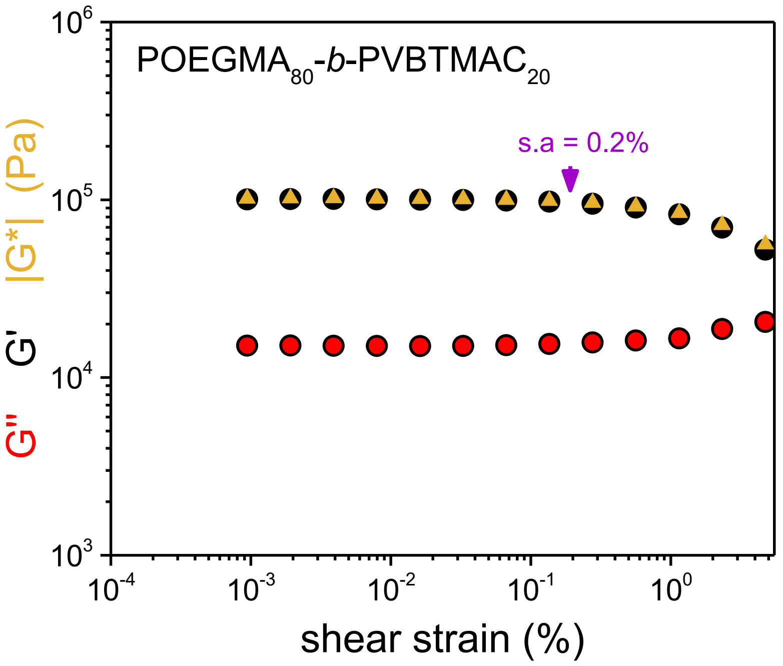

Figure S1. Absolute value of the shear storage modulus as a function of the shear strain amplitude presented on a log-log scale. The vertical arrow shows the used strain amplitude into the linear viscoelastic regime.

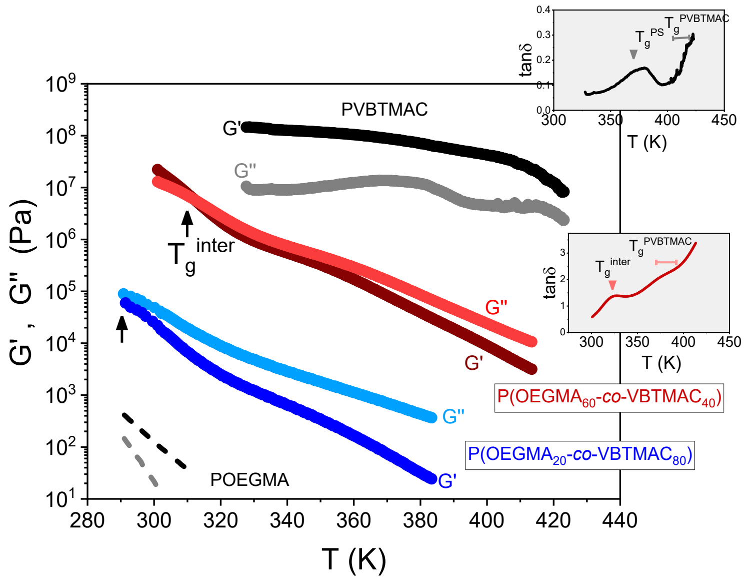

Figure S2. Temperature dependence of the storage and loss shear moduli for the PVBTMAC homopolymer (black symbols), P(OEGMA$_{60}$-*co*-VBTMAC$_{40}$) (red symbols) and P(OEGMA$_{80}$-*co*-VBTMAC$_{20}$) (blue symbols) upon heating with a rate of 5 K·min^{-1}. **(Insets)** Loss factor aa a function of temperature. The vertical arrows and horizontal lines indicate the interfacial and PVBTMAC glass transition temperatures, respectively.

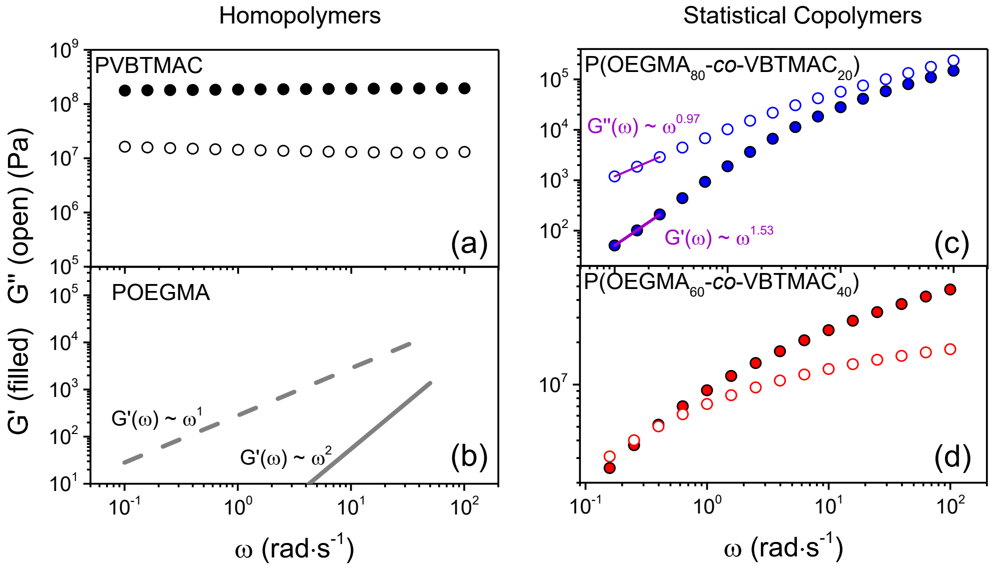

Figure S3. Angular frequency dependence of the storage (filled symbols or solid lines) and loss (open symbols or dashed lines) shear moduli for (a) PVBTMAC, (b) POEGMA, (c) P(OEGMA$_{80}$-*co*-VBTMAC$_{20}$) and (d) P(OEGMA$_{60}$-*co*-VBTMAC$_{40}$), at a temperature of 293 K.

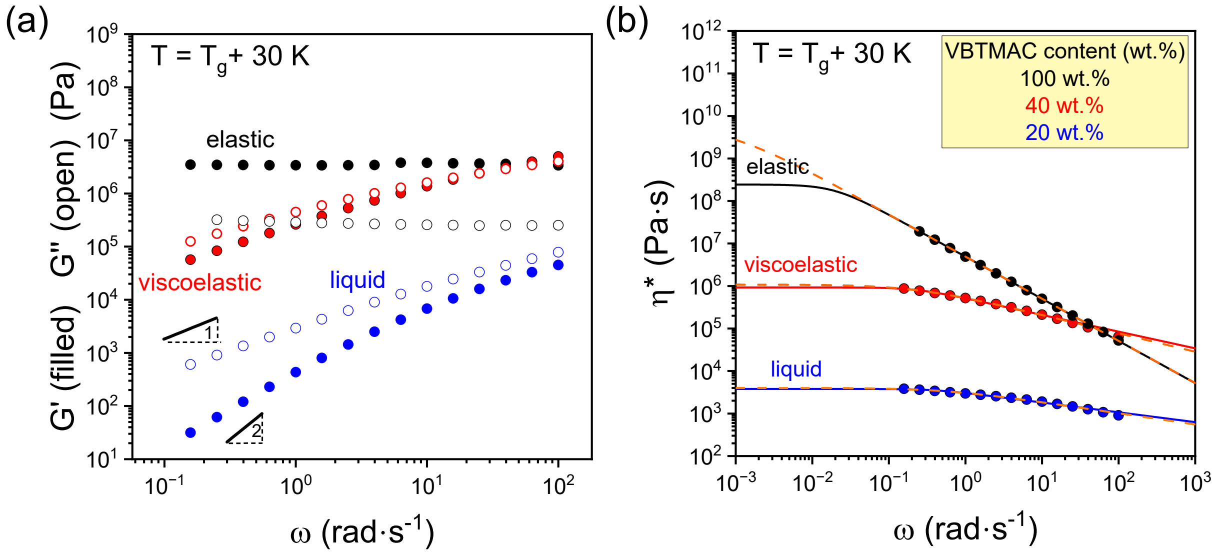

Figure S4. Angular frequency dependence of **(a)** the storage (filled symbols) and loss (open symbols) shear moduli and **(b)** complex viscosity of P(OEGMA$_{80}$-*co*-VBTMAC$_{20}$) (blue symbols), P(OEGMA$_{60}$-*co*-VBTMAC$_{40}$) (red symbols) and the PVBTMAC homopolymer (black symbols) at a temperature of 293 K. Lines with slopes 1 and 2 are also shown in **(a)**. The solid and dashed lines in **(b)** represent fits by Eq. 1 and Eq. 2 of the main manuscript, respectively.

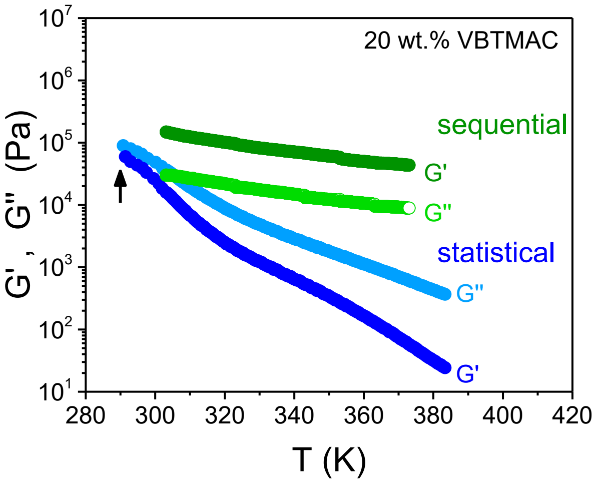

Figure S5. Temperature dependence of the storage (filled symbols) and loss (open symbols) shear moduli for P(OEGMA$_{80}$-*co*-VBTMAC$_{20}$) (blue symbols) and POEGMA$_{80}$-*b*-VBTMAC$_{20}$ (green symbols) upon heating with a rate of 5 K·min^{-1}.

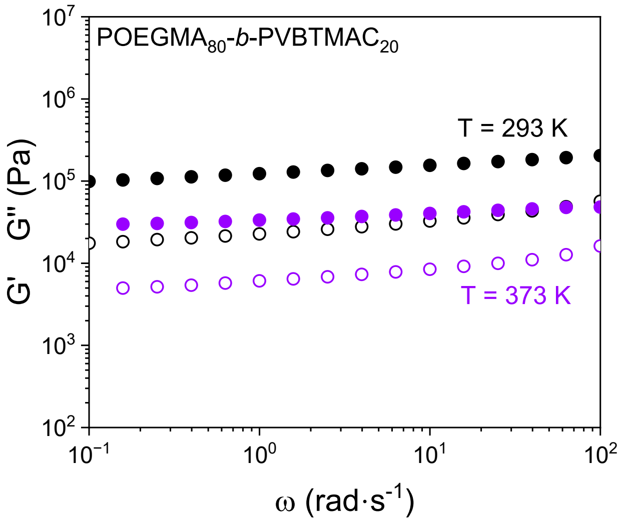

Figure S6. Dynamic frequency sweeps for the diblock copolymer with 20 wt.% of PVBTMAC at the temperatures T = 293 K (black) and T = 373 K (purple).

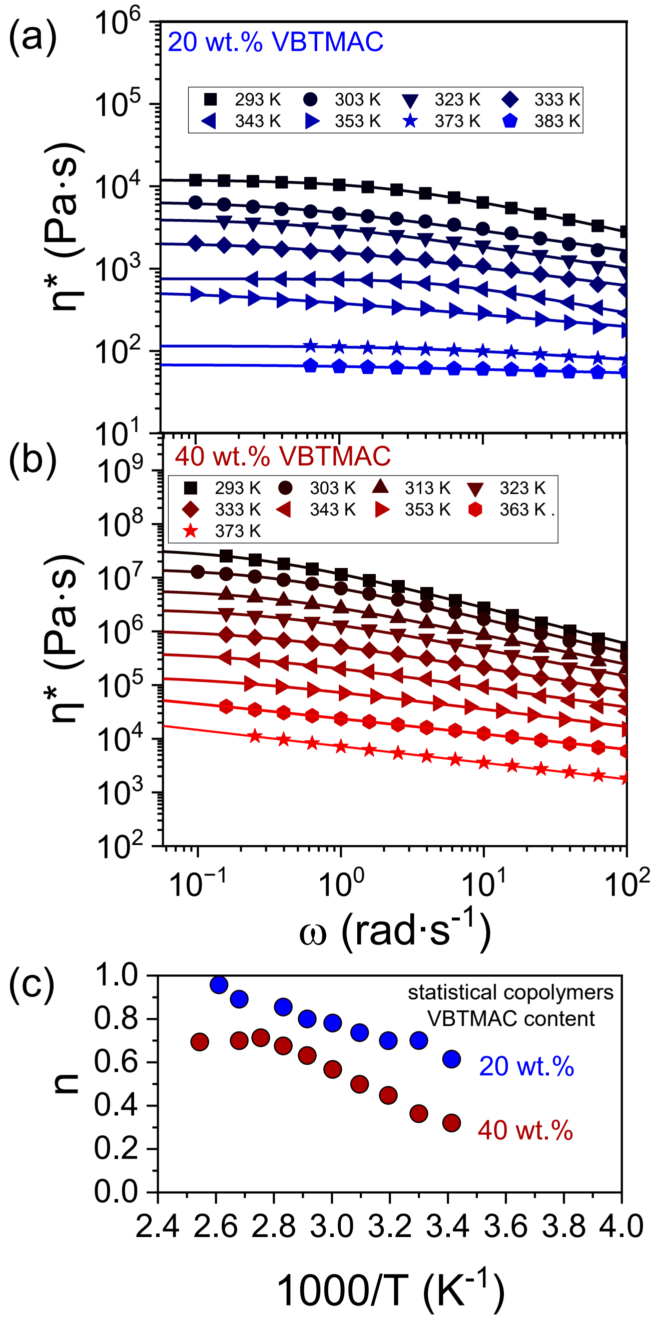

Figure S7. Angular frequency dependence of complex viscosity for **(a)** P(OEGMA$_{80}$-*co*-VBTMAC$_{20}$) and **(b)** P(OEGMA$_{60}$-*co*-VBTMAC$_{40}$) at different temperatures. The solid lines represent fits by the CM model (Eq. 2). **(c)** Inverse temperature dependence of the extracted power law index values.

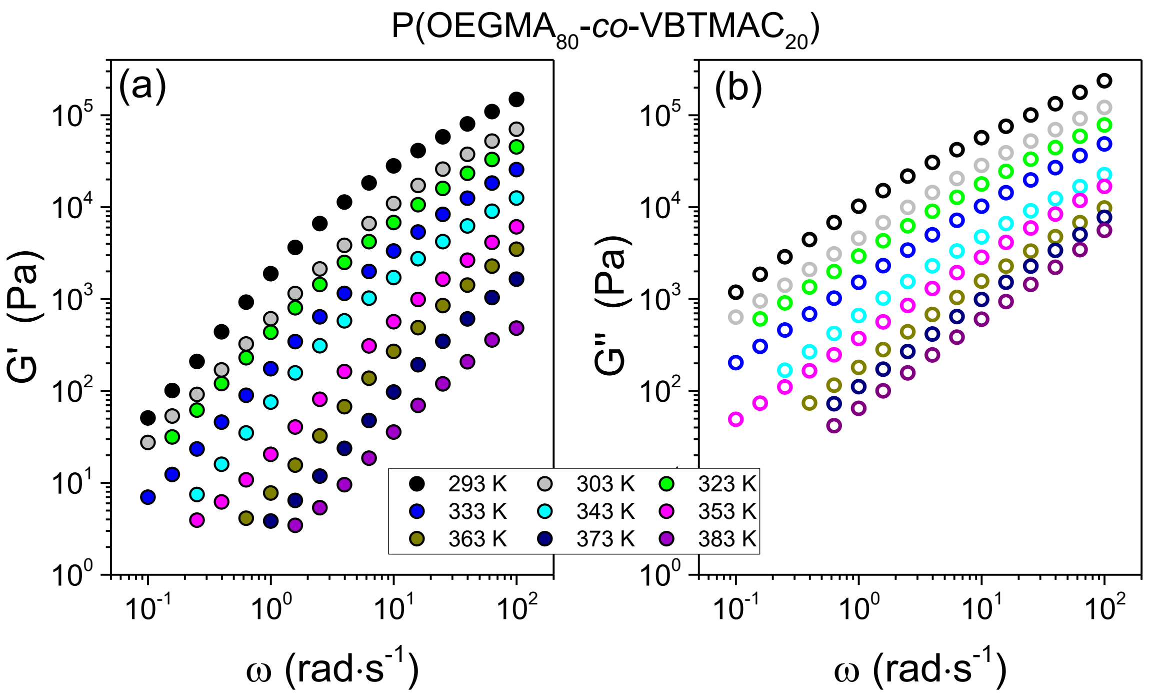

Figure S8. Angular frequency dependence of **(a)** storage and **(b)** loss moduli for the P(OEGMA$_{80}$-*co*-VBTMAC$_{20}$) statistical copolymer at the temperatures; 293 K (black), 303 K (gray), 323 K (green), 333 K (blue), 343 K(cyan), 353 K (magenta), 363 K (dark-yellow), 373 K (navy) and 383 K (purple), as indicated.

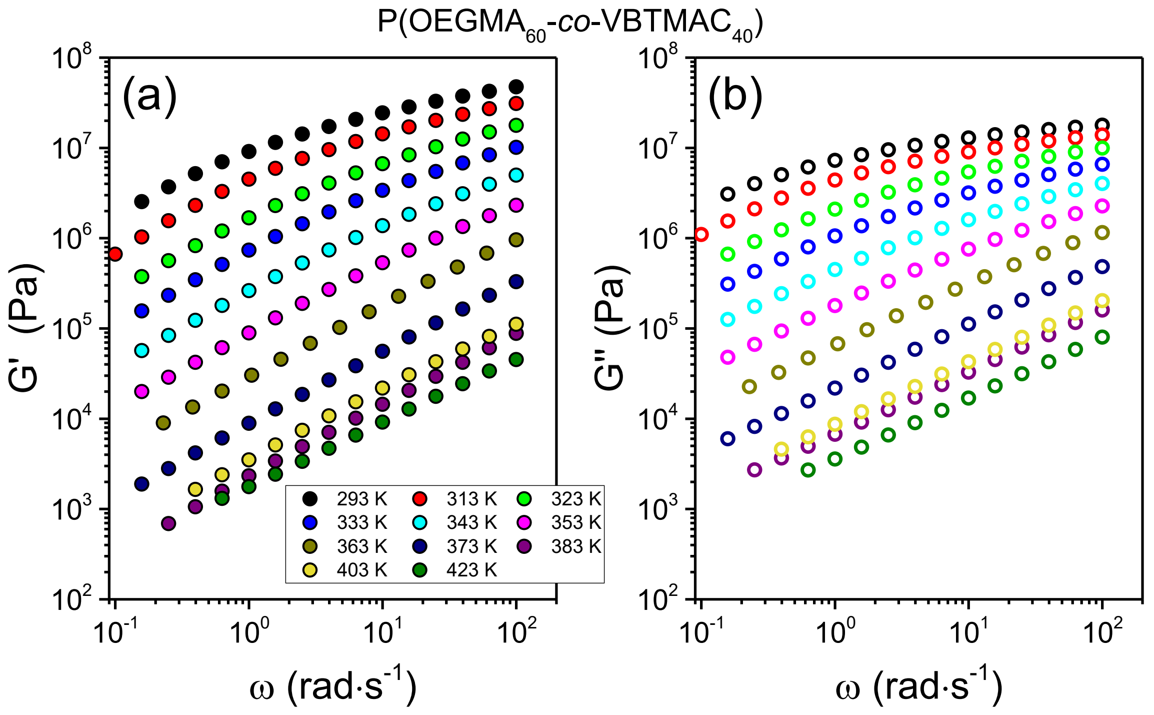

Figure S9. Angular frequency dependence of shear **(a)** storage and **(b)** loss moduli for the statistical copolymer with 40 wt.% VBTMAC content at the temperatures 293 K (black), 313 K (red), 323 K (green), 333 K (blue), 343 K(cyan), 353 K (magenta), 363 K (dark-yellow), 373 K (navy), 383 K (purple), 403 K (yellow) and 423 K (olive).

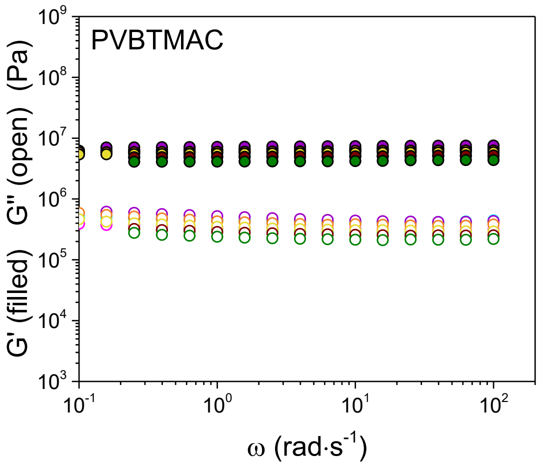

Figure S10. Angular frequency dependence of shear **(a)** storage (filled symbols) and loss moduli (open symbols) for the PVBTMAC homopolymer, at the temperatures 323 K (green), 343 K(cyan), 353 K (magenta), 363 K (dark yellow), 383 K (purple), 393 K (orange), 403 K (yellow), 413 K(wine) and 423 K (olive).

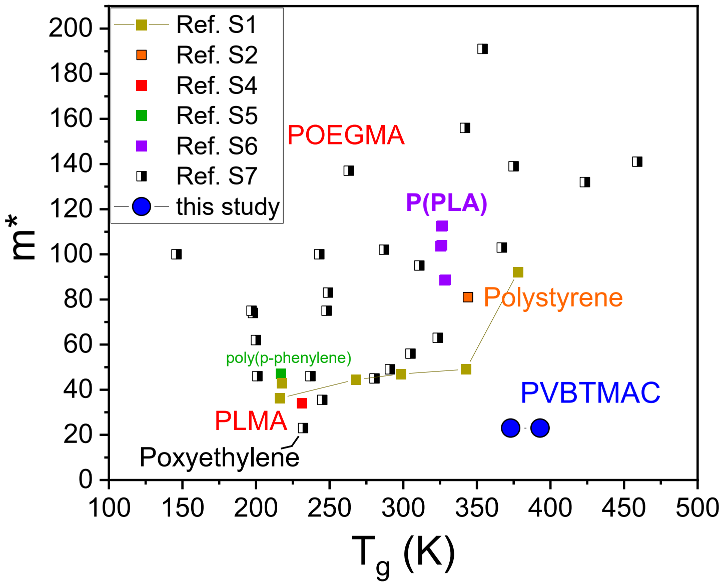

Figure S11. Fragility at T_g as a function of glass transition temperature for the PVBTMAC homopolymer (blue symbols). Data from common polymers are included, taken from Refs [S1, S2-S7], as indicated.

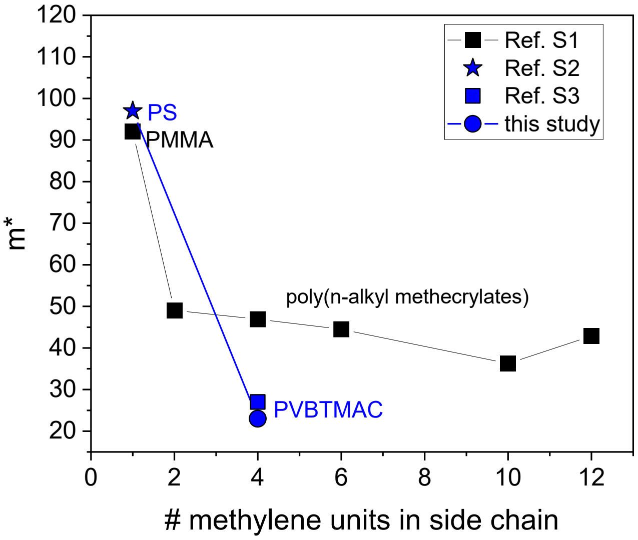

Figure S12. Fragility, *m* , plotted as a function of the number of methylene units on the side chain of polystyrene (blue star), PVBTMAC (blue circle and square) and the family of poly(*p*-alkyl methacrylates) (black symbols), taken from Refs [S1-S3].

References

(S1) Floudas, G.; Štepánek, P. Structure and dynamics of poly (n-decyl methacrylate) below and above the glass transition. *Macromolecules* **1998**, *31* (20), 6951-6957.
(S2) Pipertzis, A.; Hossain, M. D.; Monteiro, M. J.; Floudas, G. Segmental dynamics in multicyclic polystyrenes. *Macromolecules* **2018**, *51* (4), 1488-1497.
(S3) Pipertzis, A.; Chroni, A.; Pispas, S.; Swenson, J. Molecular Dynamics and Self-Assembly in Double Hydrophilic Block and Random Copolymers. *The Journal of Physical Chemistry B* **2024**, *128* (45), 11267-11276.
(S4) Pipertzis, A.; Skandalis, A.; Pispas, S.; Floudas, G. Nanophase Segregation Drives Heterogeneous Dynamics in Amphiphilic PLMA-b-POEGMA Block-Copolymers with Densely Grafted Architecture. *Macromolecular Chemistry and Physics* **2024**, *225* (19), 2400180.
(S5) Mierzwa, M.; Floudas, G.; Neidhöfer, M.; Graf, R.; Spiess, H. W.; Meyer, W. H.; Wegner, G. Constrained dynamics in supramolecular structures of poly (p-phenylenes) with ethylene oxide side chains: A combined dielectric and nuclear magnetic resonance investigation. *The Journal of chemical physics* **2002**, *117* (13), 6289-6299.
(S6) Hu, M.; Xia, Y.; McKenna, G. B.; Kornfield, J. A.; Grubbs, R. H. Linear rheological response of a series of densely branched brush polymers. *Macromolecules* **2011**, *44* (17), 6935-6943.
(S7) Qin, Q.; McKenna, G. B. Correlation between dynamic fragility and glass transition temperature for different classes of glass forming liquids. *Journal of Non-Crystalline Solids* **2006**, *352* (28-29), 2977-2985.
